# Experimental Investigation and Bayesian Assessment for Permeability Characteristics of Lightweight Ceramsite Concrete

**DOI:** 10.3390/ma17164112

**Published:** 2024-08-20

**Authors:** Min Li, Yongjun Wang, Mengzhang Chen, Lin Zhang, Yinshan Xu, Hongbo Zhao, Jiaolong Ren

**Affiliations:** 1 School of Civil Engineering and Geomatics, Shandong University of Technology, Zibo 255000, China; 22507020009@stumail.sdut.edu.cn (M.L.); 23507030871@stumail.sdut.edu.cn (Y.W.); 23507030882@stumail.sdut.edu.cn (M.C.); kennylzh@connect.hku.hk (L.Z.); hbzhao@sdut.edu.cn (H.Z.); 2 Zhejiang Scientific Research Institute of Transport, Hangzhou 310039, China; xys0613@126.com

**Keywords:** lightweight ceramsite concrete, ceramsite aggregate size, ceramsite aggregate content, permeability characteristics, Bayesian approach

## Abstract

Ceramsite concrete is one of the most widely used lightweight concretes at present. Although mechanical properties of ceramsite concrete have been extensively discussed, its permeability characteristics are neglected in previous studies. Considering the importance of permeability resistance to concrete, the permeability grade and residual compressive strength after permeability of ceramsite concrete are analyzed in this study. The influence of ceramsite content and size on the permeability grade and residual strength of ceramsite concrete were investigated by the orthogonal experimental method. To further understand the above influence, an improved Bayesian framework for small sample data is also established to analyze the permeability grade and residual strength. Results show that the water–binder ratio and the content of 20–30 mm ceramsite aggregates are the most and least significant influencing factors affecting the permeability characteristics, respectively. The 5–10 mm and 10–20 mm ceramsite aggregates play secondary roles. Increasing 5–10 mm and 10–20 mm ceramsite aggregates is not helpful for improving the permeability resistance of ceramsite concrete. Compared with the orthogonal method, the proposed Bayesian framework is a useful tool for revealing the effects of various factors, which can cut the time cost and provide parameter visualization for the analysis process. Results show that the permeability resistance and residual strength of ceramsite concrete are improved significantly under optimal conditions. The permeability grade and residual strength are increased 200% and 80.3%, respectively. In addition, the residual strength may be more suitable for evaluating the permeability characteristics than the permeability grade.

## 1. Introduction

Concrete is the most widely used construction material in the field of civil engineering. Currently, many new types of concrete had been developed to meet the increasing demands of contemporary structures. Owing to their advantages, such as high strength, light weight, good stability, environmental protection, and low costs, lightweight aggregate concretes are widely used in bridges and high-rise building structures [1,2,3]. Ceramsite concrete is a kind of lightweight concrete that partially replaces natural aggregates using ceramsite aggregates [4,5]. Compared with ordinary concrete, the self-weight of the ceramsite concrete could be reduced by more than 20% under the premise of maintaining strength.

The ceramsite is a porous granular composite material obtained from natural minerals and industrial waste materials [6]. It has the advantages of large specific surface area, low thermal conductivity, good thermal insulation performance, wide source, and mature industrialization [7]. Owing to the great difference between ceramsite aggregates and natural aggregates, there is a significant difference between the performance of ceramsite concrete and ordinary concrete. Hence, the performance of ceramsite concrete has been widely investigated in recent years.

For mechanical properties, Zang et al. [8] and Chen et al. [9] prepared dehydrated silt ceramsite concrete and fly ash ceramsite concrete, respectively, to reveal the law of ceramsite concrete strength. Han et al. [10] and Bu et al. [11] analyzed the effect of the water–cement ratio on the mechanical properties of ceramsite concrete. Zhu et al. [12] studied the flexural-bearing performance of ceramsite concrete beams after creep, which provided a theoretical basis for creep design of lightweight aggregate structures. Wu et al. [13] and Wang et al. [14] investigated the damage evolution characteristics of ceramsite concrete using split Hopkinson pressure bar and acoustic emission monitoring. Wu et al. [13] adopted a modified split Hopkinson pressure bar test to detect the mechanical response of shale ceramsite concrete, followed by CT scanning and ultrasonic testing. De Maio et al. [15] proposed an advanced numerical model to simulate the fracture propagation of concrete based on the cohesive zone method and moving grid technique under mixed-mode fracturing, which provided an effective and a reliable predicting effect.

For durability, Li et al. [3], Yuan et al. [16], and Zeng et al. [17,18] investigated the freezing resistance of ceramsite concrete after different freezing and thawing cycles. Deng et al. [2], Shen et al. [19], and Zhang et al. [20] analyzed the drying–wetting shrinkage characteristics of ceramsite concrete. Yao et al. [21] evaluated the fire resistance of ceramsite concrete at high-temperatures. Zhang et al. [22] proved that the rough surface of the ceramsite could improve the bonding ability between ceramsite and cement mortar to enhance the strength of ceramsite concrete. Gao et al. [23] found that the permeability of ceramsite concrete was better than ordinary concrete, mainly owing to the porosity of ceramsite aggregates being higher than natural aggregates. Fan et al. [24] found that the permeability of ceramsite concrete decreased with an increase in curing temperature, while the heat preservation strength increased accordingly. The above studies all indicated that there was significant difference in durability between ceramsite concrete and ordinary cement concrete, although ceramsite aggregates only partially replaced natural aggregates.

Permeability resistance was one of the most important factors influencing the durability of lightweight concrete [25,26,27]. For instance, Palanisamy et al. [25], Bulut [26], and Chao et al. [27] investigated the permeability resistance of lightweight concrete using different lightweight aggregates (shell aggregates, perlite aggregates, and plastic waste aggregates), and all indicated that permeability characteristics had significant difference among different types of concretes. The existing studies [28,29] also proved that the difference in lightweight aggregates plays a significant role in the performance of lightweight concrete. Obviously, it could be speculated that the permeability resistance of ceramsite concrete was consequentially different from other lightweight concretes because of the difference between ceramsite aggregates and other lightweight aggregates. Hence, it was necessary to systematically investigate the permeability resistance of ceramsite concrete. However, as in the above literature review, it can be found that the permeability resistance of ceramsite concrete was less discussed in exciting studies [2,3], although mechanical properties and other durabilities of ceramsite concrete were extensively analyzed. Only Gao et al. [23] and Fan et al. [24] involved the topic of the permeability resistance of ceramsite concrete. However, they neglected the effects of compositions (e.g., ceramsite content, ceramsite size, admixtures) on permeability resistance of ceramsite concrete.

Hence, this study mainly focused on the effects of material composition (e.g., ceramsite content, ceramsite size, water–binder ratio, admixtures) on permeability resistance of ceramsite concrete from a view of permeability grade and residual compressive strength after permeability test. Moreover, a Bayesian framework is further established to analyze the permeability grade and residual strength to estimate these factors more comprehensively.

## 2. Materials and Methods

### 2.1. Materials

The ceramsite concrete was prepared by cement, ceramsite aggregates, natural aggregates, sands, fly ash, silica fume, and water-reducing agent.

(1) Cement

Ordinary Portland cement P.O42.5 was adopted in this study, of which the technical properties are listed in Table 1.

(2) Ceramsite aggregates

**Figure 1:**
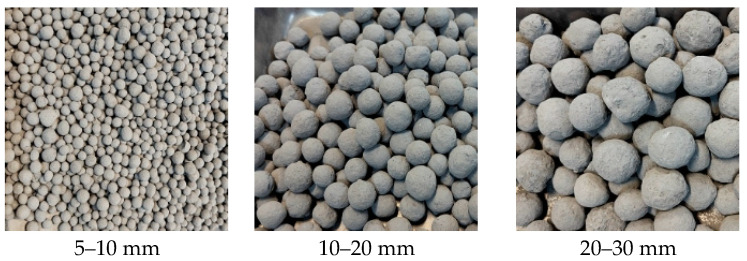
Powdery ash ceramide.

Pulverized coal ash ceramsites produced by Zhejiang Ningbo Zhongjin Environmental Protection Technology Co., Ltd. (Ningbo, China) were selected, as shown in Figure 1. The ceramsite aggregates were divided into three sizes: 5–10 mm, 10–20 mm, and 20–30 mm. Their technical properties are presented in Table 2. It should be explained that the aggregates were generally divided into four groups: 20–30 mm, 10–20 mm, 5–10 mm, and 0–5 aggregates in China’s engineering practice. The first three were coarse aggregates, and the last one was the fine aggregates. Because this study mainly aimed to adopt ceramsite particles to replace coarse aggregates, the three sizes (20–30 mm, 10–20 mm, and 5–10 mm) of ceramsite aggregates were selected.

(3) Natural aggregates

**Figure 2:**
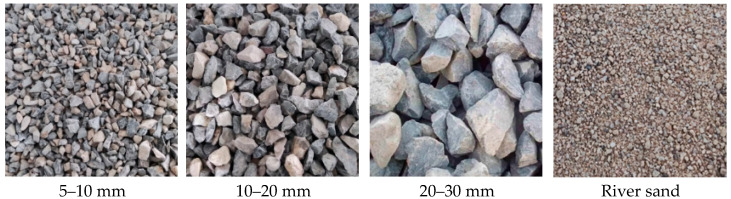
Aggregates with different particle sizes.

Basalt aggregates obtained from Zibo were adopted in this study, which were divided into three sizes: 5–10 mm, 10–20 mm, and 20–30 mm, as shown in Figure 2. The sand was river sand. The technical properties of the aggregates and river sand are presented in Table 3 and Table 4, respectively.

(4) Fly ash and silica fume

Fly ash (grade II) and silica fume produced by Hebei Boheng Minerals Co., Ltd. (Hengshui, China) were selected, of which the technical properties are presented in Table 5 and Table 6, respectively.

(5) Water-reducing agent

A polycarboxylic acid water-reducing agent produced by Laiyang Hongxiang building admixture Co., Ltd. (Yantai, China) was selected, of which the technical properties are presented in Table 7.

### 2.2. Method

#### 2.2.1. Laboratory Test

**Figure 3:**
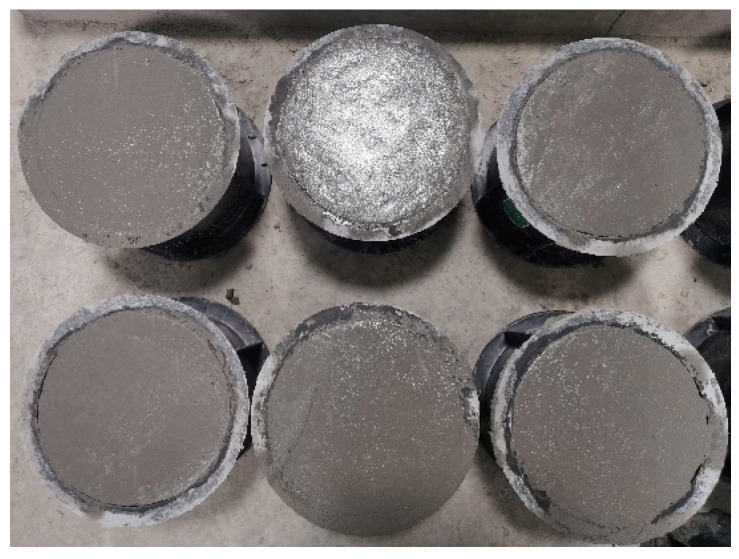
Permeability characteristics experimental sample.

In this study, the permeability characteristics of ceramsite concrete were evaluated by permeability grade and osmotic pressure. All experiments were conducted in accordance with the Chinese test standard “Testing Method of Cement and Concrete for Highway Engineering (JTG 3420-2020)” [31]. The detailed experiment procedure was as follows:

- Cylinder samples with the size of *Φ* 150 mm × *H* 150 mm were prepared according to the standard method, as shown in Figure 3.
- After the curing age of 28 d, a layer of melted sealing material was rolled on the side of the sample. Immediately, the concrete sample should be pressed into the preheated test mold, of which the bottom surface must be level with the bottom of the test mold.
- After the test mold was cooled, the sample was loaded by water pressure that started from 0.1 MPa and increased by 0.1 Mpa for every 8 h. Six samples should be simultaneously tested.
- The surface of the samples should be observed hourly until water permeated from the surface of three samples in the six samples. At the same time, the test could be stopped after recording the water pressure. The permeability grade could be calculated by Equation (1). It should be explained that the permeability grade only had six levels: P2, P4, P6, P8, P10, and P12. The maximum permeability grade is P12. If the water pressure was increased to 1.2 Mpa and the water did not permeate from the third sample after 8 h, the permeability grade could be recorded by P12.

(1) P=10H−1

where *P* was the permeability grade and *H* was the recorded water pressure.

- After finishing the permeability tests, the samples were subjected to unconfined compressive strength tests to investigate the residual mechanical properties.

#### 2.2.2. Bayesian Method

The Bayesian method could acquire the relation of material composition and performance index based on experimental data by choosing one optimal model class from a set of candidate model classes [32], which had the advantages in higher robustness and better fitting degree in contrast to conventional probabilistic methods [33]. However, the existing Bayesian method relied on the condition of large sample data. In this study, the “likelihood” was employed as the criterion for model class selection, which represented the desired balance between the robustness and fitting degree, to establish an improved Bayesian framework for small sample data. The likelihood of one group data L for a Bayesian model class Ci can be expressed by Equation (2):

(2) $$P\left(C_{i}|L,J\right)=\frac{\overset{\mathrm{Evidence}}{\overset{︷}{P\left(L|C_{i},J\right)}}\overset{\mathrm{Prior}}{\overset{︷}{P\left(C_{i}|J\right)}}}{P\left(L|J\right)};i=1,2,\ldots N_{C}$$

where $P\left(C_{i}|J\right)$ stands for the assessment of the initial rationality of both the *C_i_* model class and the $\sum_{i=1}^{N_{C}}P\left(C_{i}|J\right)=1$ model class. *J* stands for user’s subjective judgment based on professional knowledge. $P\left(L|J\right)=\sum_{i=1}^{N_{C}}P\left(L|C_{i},J\right)P\left(C_{i}|J\right)$ stands for a regularized denominator factor for each model class. $P\left(L|C_{i},J\right)$ stands for the basis for the given data *L* of Bayesian model class *C_i_*. Higher values of the $P\left(L|C_{i},J\right)$ mean higher reliability. Building on the research of Papadimitriou et al. [34], Beck and Yuen [35] proposed the estimation of $P\left(L|C_{i},J\right)$, as shown in Equation (3).

(3) $$\overset{\mathrm{Evidence}}{\overset{︷}{P\left(L|C_{i},J\right)}}\approx \overset{\mathrm{Likelihood}\;\mathrm{function}}{\overset{︷}{P\left(L|\hat{\zeta },C_{i}\right)}}\overset{\mathrm{Ockham}\;\mathrm{factor}}{\overset{︷}{p\left(\hat{\zeta }|C_{i}\right){\left(2\pi \right)}^{N_{i}/2}{\left|H_{i}\left(\hat{\zeta }\right)\right|}^{-\frac{1}{2}}}};i=1,2,\ldots ,N_{C}$$

where $N_{i}$ expresses the number of uncertain parameters in the model class $C_{i}$. $\zeta_{i}$ is the parameter vector for the $C_{i}$ model class. ${\hat{\zeta }}_{i}$ is equal to the most probable value of $\zeta_{i}$. $P\left(L|{\hat{\zeta }}_{i},C_{i}\right)$ and $P\left({\hat{\zeta }}_{i}|C_{i}\right){\left(2\pi \right)}^{N_{i}/2}{\left|H_{i}\left({\hat{\zeta }}_{i}\right)\right|}^{-\frac{1}{2}}$ are the likelihood function and Ockham factor [29], respectively, which represent the fitting degree and robustness of the $C_{i}$ model class to the data at ${\hat{\zeta }}_{i}$. $H_{j}\left({\hat{\zeta }}_{j}\right)$ is the Hessian matrix of $-\ln\left[P\left(L|\zeta_{i},C_{i}\right)p\left(\zeta_{i}|C_{i}\right)\right]$. A higher Ockham factor indicates better robustness of the model class.

Thus, the Bayesian prediction equation is as follows:

(4) $$F=f+\varepsilon =\theta^{T}z+\varepsilon =\theta_{0}z_{0}+\theta_{1}z_{1}+\theta_{2}z_{2}+\ldots +\theta_{N_{x}}z_{N_{x}}+\varepsilon$$

where ***F*** and *f* represent the measured and predicted values of the target output index (permeability grade and residual compressive strength in this study), respectively. *ε* is the prediction error (both modeling and measurement error). *θ* and *z* are column vectors, which contain uncertain fitting coefficients and measured values, respectively. Typically, *z*_0_ represents a constant term equal to 1. In this study, *z* comprises various combinations of concrete mixture normalization parameters, including the water–binder ratio, fly ash, silica fume, the CRR of 5–10 mm ceramsite aggregates, the CRR of 10–20 mm ceramsite aggregates, and the CRR of 20–30 mm ceramsite aggregates. The prediction error assumes that the Gaussian random variable has zero mean and zero variance to control the variance of the error. Thus, *ζ* can be expressed as follows:

(5) $$\zeta ={\left[\theta^{T},\;\sigma_{\varepsilon }^{2}\right]}^{T}$$

It is necessary to identify the uncertain model parameters in advance before selecting the model class. $\hat{\zeta }$ corresponds to the maximum $p\left(L|{\hat{\zeta }}_{i},C_{i}\right)$, which means the goodness-of-fit is the best for the given data. Hence, ${\hat{\theta }}_{i}$ can be calculated as follows:

(6) $${\hat{\theta }}_{i}=\frac{\partial J_{g}\left(\theta |L,C_{i}\right)}{\partial \theta_{k}}=0;k=1,2,\ldots ,N_{i}$$

(7) $$J_{g}\left(\theta |L,C_{i}\right)=\frac{1}{N}{\sum_{n=1}^{N}\left[f_{\left(n\right)}\left(z_{\left(n\right)};\theta ,C_{i}\right)-F_{\left(n\right)}\right]}^{2}$$

(8) $${\hat{\sigma }}_{\varepsilon }^{2}=\underset{\theta }{\min}\left[J_{g}\left(\theta |L,C_{i}\right)\right]=J_{g}\left(\hat{\theta }|L,C_{i}\right)$$

where $N_{i}$ stands for the number of parameters of the Bayesian-based prediction equation for the model class $C_{i}$. $J_{g}\left(\theta |L,C_{i}\right)$ is the goodness-of-fit function.

The probability density function (PDF) of updated $\zeta^{t+1}$ for the given data *L* and model class $C_{i}$ is as follows:

(9) $$P\left(\zeta^{t+1}|L,C_{i}\right)=c_{0}P\left(\zeta^{t}|C_{i}\right)\overset{p\left(L|\theta ,C_{i}\right)=\;\mathrm{likelihood}\;\mathrm{function}}{\overset{︷}{{\left(2\pi \right)}^{-\frac{N}{2}}{\hat{\sigma }}_{\varepsilon }^{-N}\exp\left[-\frac{N}{2\sigma_{\varepsilon }^{2}}J_{g}\left(\theta |L,C_{i}\right)\right]}}$$

where *c*_0_ is a normalized constant, so that $P\left(\zeta |L,C_{i}\right)$ is the unit volume. $P\left(\zeta |C_{i}\right)$ is the prior PDF, which represents the user’s initial identification of the rationality of the fitting parameters of the model. For convenience, a uniform prior PDF can be used.

## 3. Permeability Characteristics

### 3.1. Orthogonal Experimental Design

Owing to the complex composition of ceramsite concrete, an orthogonal method was used to design the experiments to investigate the permeability characteristics of ceramsite concrete. The experimental factor and the corresponding experimental level are presented in Table 8. The experimental sequence can be found in Table 9. The ceramsite replacement ratio (CRR) was equal to the ratio of ceramsite aggregate volume to total aggregate volume (both ceramsite aggregates and natural aggregates). The test results are presented in Table 10.

### 3.2. Test Results and Analysis

**Figure 4:**
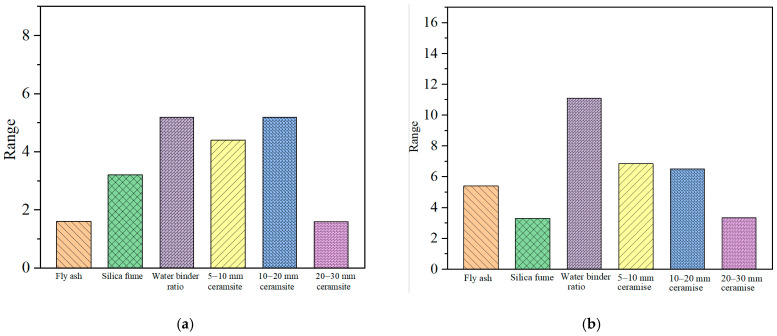
Ranges of different factors: (**a**) Permeability grade. (**b**) Residual strength.

As previously mentioned, the permeability grade and residual strength after permeability were used to evaluate the permeability characteristics of lightweight ceramsite concrete. The orthogonal analysis results [22,23] are presented in Table 11. The larger the range, the more significant the influence of the factor on the performance. The range of each influencing factor was shown in Figure 4. The range could be calculated using Equation (10):

(10) $$Range=TD_{\max}-TD_{\min}$$

where *TD*_max_ and *TD*_min_ = the maximum and minimum value of the average test data.

As shown in Figure 4, it can be found that:

(1) The water–binder ratio and the CRR of 10–20 mm ceramsite aggregates have the highest influence on the permeability grade, followed by the CRR of 5–10 mm ceramsite aggregates and silica fume. The fly ash and the CRR of 20–30 mm ceramsite aggregates have the lowest influence on the permeable grade.

(2) For residual strength, although the water–binder ratio is still the most significant factor, the influence degrees of other factors are different from the permeability grade. The range of the silica fume, the CRR of 5–10 mm ceramsite aggregates, and the CRR of 10–20 mm ceramsite aggregates on the residual strength are obviously lower than the permeability. The range of the silica fume is even the lowest for the residual strength. Moreover, the range of fly ash significantly increases from the lowest one for the permeability grade to the fourth-highest one for the residual strength. This is due to the fact that the permeability grade can only be an even number, such as 2, 4, …, 12, according to the Chinese specification [31]. In this case, the range of the permeability grade is different from that of the residual strength. In addition, the permeability grade reflects the physical properties of concrete, while the residual strength is the mechanical properties. The above results show that there are differences between the physical properties and the mechanical properties after permeation.

**Figure 5:**
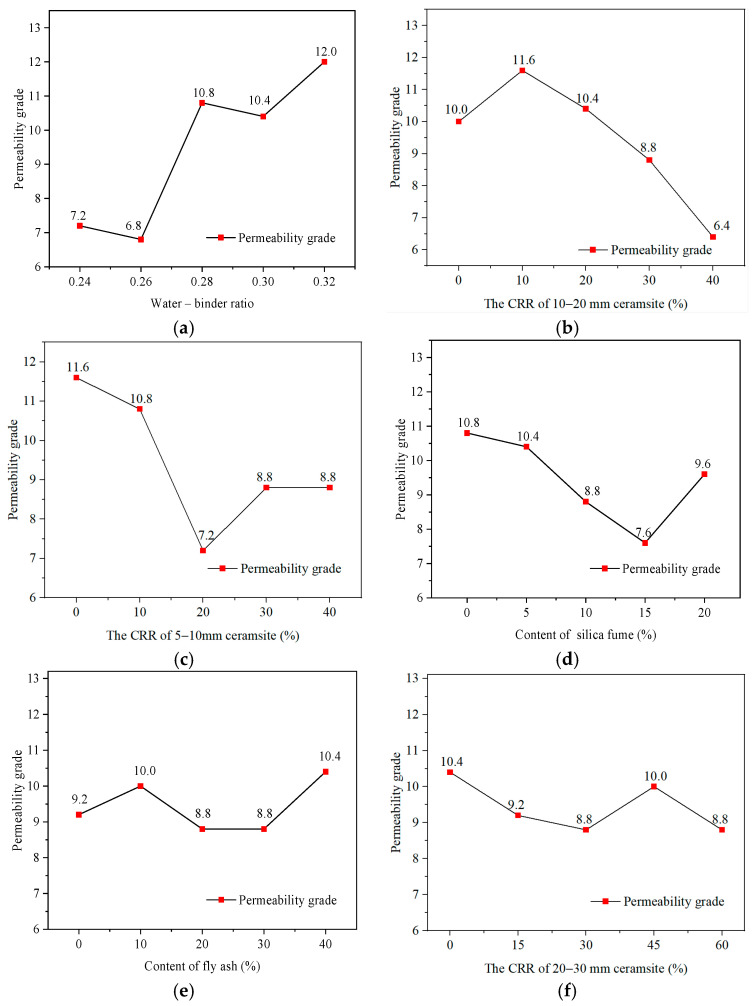
The influence trend of factors on permeability grade: (**a**) Water–binder ratio. (**b**) The CRR of 10–20 mm ceramsite aggregates. (**c**) The CRR of 5–10 mm ceramsite aggregates. (**d**) Silica fume content. (**e**) Fly ash content. (**f**) The CRR of 20–30 mm ceramsite aggregates.

**Figure 6:**
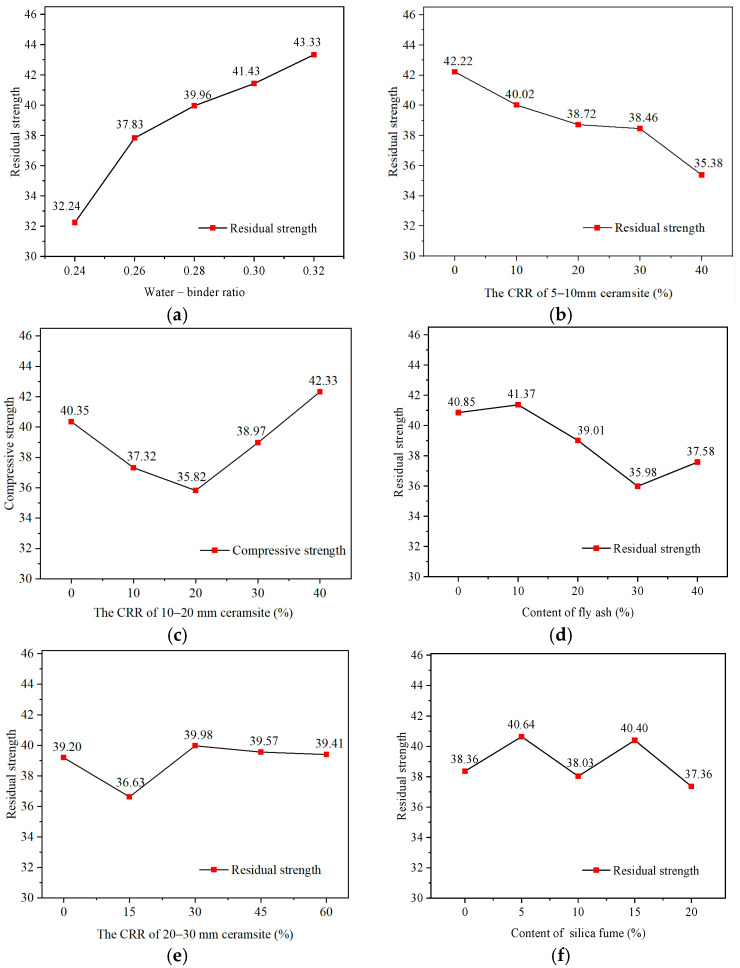
The influence trend of factors on residual strength. (**a**) Water–binder ratio. (**b**) The CRR of 5–10 mm ceramsite aggregates. (**c**) The CRR of 10–20 mm ceramsite aggregates. (**d**) Fly ash content. (**e**) The CRR of 20–30 mm ceramsite aggregates. (**f**) Silica fume content.

The influence trends of the experimental factors under different experimental levels are shown in Figure 5 and Figure 6.

According to Figure 5 and Figure 6, the following conclusions can be drawn:

- As previously mentioned, the water–binder ratio is the most important factor for the permeability characteristics. In general, the permeability grade and residual strength increase with the increase in the water–binder ratio. This is due to the fact that cement is the most important component for the strength and densification of ceramsite concrete. It should be explained that in Figure 6a, there can be some fluctuation in the curve. It is due to the fact that the maximum permeability grade of concrete is P12 according to the Chinese test standard [31]; however, the actual permeability grade of some concretes may be higher than P12. It will affect the actual relationship between the permeability grade and water–binder ratio. Just for this, the residual compressive strength after the permeability test is adopted to indirectly characterize the permeability resistance in this study.
- Compared to the CRR of 20–30 mm ceramsite aggregates, the CRR of 5–10 mm and 10–20 mm ceramsite aggregates are the secondary significant influencing factors. This is as a result of the higher water absorption rates of the 5–10 mm and 10–20 mm ceramsite aggregates, owing to their larger specific surface areas. The study of Kong et al. [28] also supported this conclusion, which shows that the higher water absorption of ceramsite can change the performance of ceramsite concrete.
- The permeability grade and residual strength are generally decreased with the increase in the CRR of the 5–10 mm ceramsite aggregates. It is due to the fact that the absorption characteristics of 5–10 mm ceramsite aggregates are the most significant among the three types of ceramsite aggregates, owing to their specific surface area being much larger than other ceramsite aggregates. Water permeation to a greater extent for the 5–10 mm ceramsite aggregates not only reduces the permeability grade but also will weakens the interface strength between the ceramsite aggregates and cement mortar, so as to bring some negative effects on mechanical performance. Hence, from a view of permeability characteristics, the 5–10 mm ceramsite aggregates are not suitable for preparing ceramsite concrete.
- The influence trend of the CRR of 10–20 mm ceramsite aggregates on the permeability grade is generally decreased, while the influence trend on residual strength is first decreased and then increased. It is well known that ceramsite aggregate strength plays an important role in concrete strength. Compared to the 5–10 mm ceramsite aggregates, when the 10–20 mm ceramsite aggregates reach a certain content, they can bring additional strength to concrete, owing to ceramsite aggregate strength increasing with the increase in aggregate sizes, although the permeability grade keeps going down. Hence, the content of 10–20 mm ceramsite aggregates must be strictly controlled to balance the permeability characteristics and mechanical strength when preparing ceramsite concrete.
- The change in the CRR of 20–30 mm ceramsite aggregates has less correlation to the permeability grade and residual strength. The content of 20–30 mm ceramsite aggregates can be properly improved to reduce concrete self-weight without weakening permeability characteristics.
- The effects of silica fume and fly ash are weaker than the water–binder ratio and ceramsite content (especially for 5–10 mm and 10–20 mm ceramsite aggregates). As a result, the change law of the curve is indistinctive with the change in silica fume and fly ash content. It is found that the permeability coefficient of light ceramsite is the highest, and ceramsite concrete has good permeability resistance, which is consistent with the conclusion of Gao et al. [23] and Fan et al. [24]. However, they did not study the effect of particle size on permeability resistance.
- The influence trends of these factors on permeability grade and residual strength present some commonality, showing the residual strength has potential to evaluate the permeability characteristics. Moreover, there is no absolute correlation between the permeability grade and residual strength. Higher permeability grade does not correspond to higher residual strength for ceramsite concrete. Hence, considering the importance of bearing capacity for concrete structure, the residual strength may be a better index to evaluate the permeability characteristics of ceramsite concrete.

In addition, sand content and water pressure also influence the permeability resistance of ceramsite concrete. In this study, mainly the effects of ceramsite characteristic (i.e., ceramsite size and content) and admixtures (i.e., fly ash and silica fume) on the permeability resistance are investigated. The sand rate is a fixed value (50%) which is selected according to the Chinese standard “Standard for test method of performance on ordinary fresh concrete (GB/T 50080-2016)”. Water pressure is carried out in accordance with the Chinese standard [31]. The effects of sand content and water pressure on the permeability resistance of ceramsite concrete will be analyzed in future works.

## 4. Bayesian Assessment

Considering the different combinations of influencing factors, such as the water–binder ratio (W in the following), fly ash (F in the following), silica fume (S in the following), the CRR of 5–10 mm ceramsite aggregates (CRR1 in the following), the CRR of 10–20 mm ceramsite aggregates (CRR2 in the following), and the CRR of 20–30 mm ceramsite aggregates (CRR3 in the following), a total of 63 candidate prediction models ($2^{6}-1=63$) were analyzed in the Bayesian model selection framework. PN and RN stand for the permeability grade and residual strength, respectively.

Table 12 listed all the candidate models, which are categorized based on the number of influencing factors considered. Models 1–6 considered individual factors. Models 7–21, models 22–41, models 42–56, and models 57–62 considered the combined action of two, three, four, and five influencing factors. Model 63 considered all the influencing factors (i.e., $x={\left[\begin{array}{cccc} 1 & \begin{array}{cccc} W & F & S & CRR1 \end{array} & CRR2 & CRR3 \end{array}\right]}^{T}$). Note that the constant term was adopted in the 63 models.

Prior to Bayesian analysis, the data for all variables involved in this study were normalized instead of raw data. In this study, a uniform $p\left(\theta |C_{i}\right)$ (i.e., prior probability density function) could be calculated and adopted through the min and max values of θ for each model class. The given interval must be sufficient to cover the width of the determined fit value. Table 13 presents the ranges of priors for the variables ($W_{N}$, $F_{N}$, $S_{N}$, $CRR1_{N}$, $CRR2_{N}$, and $CRR3_{N}$) listed in Table 12 after normalizing.

**Figure 7:**
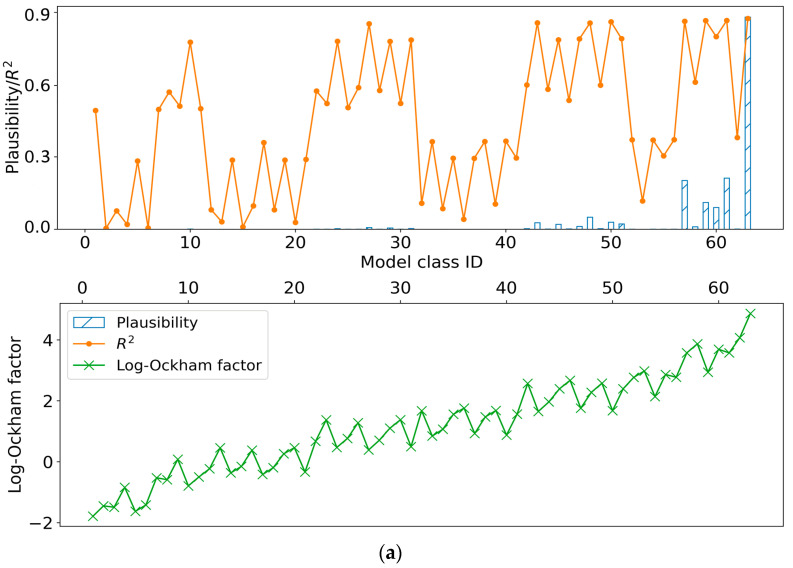

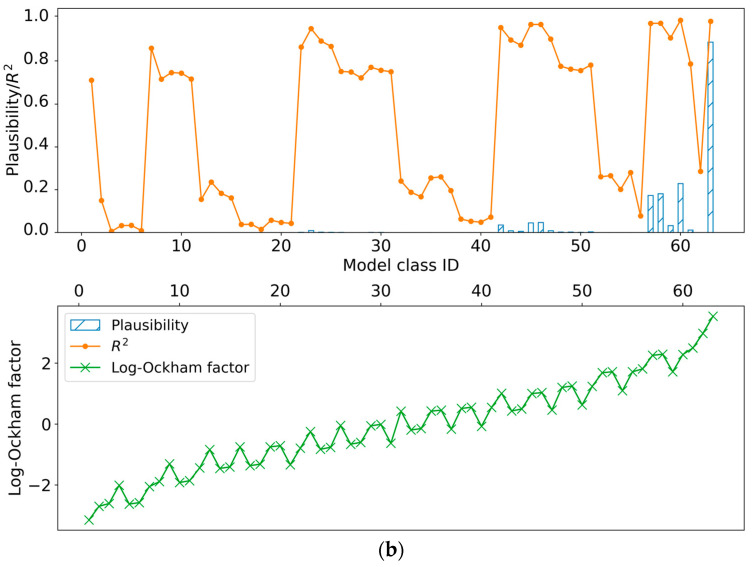
Bayesian results: (**a**) Permeability grade. (**b**) Residual strength.

Independent unified priors for the variables (see Table 12) after normalizing and the 63 model classes were used to carry out Bayesian probability analysis for the candidate model classes. The likelihoods, coefficients of determination (*R*^2^), and Log–Ockham factors of the 63 model could be found in Figure 7.

As shown in Figure 7, the highest *R*^2^ (0.891) of the models for the residual strength is larger than that (0.996) for the permeability grade. It indicates that the adaptability of these influence factors on the residual strength for the Bayesian framework is better than those on the permeability grade. However, the *R*^2^ values and plausibility of model 63 are both highest among all the models for both the permeability grade and residual strength, indicating that model 63 is the best-fitting model of all the candidate models. This is reasonable due to the fact that there are the highest-fitting coefficients and the greatest number of variables in model 63.

Moreover, there are eight models (27, 43, 48, 50, 57, 59, 61, and 63) which have higher *R*^2^ (over 0.85) for the permeability grade. In other words, the prediction results of the eight models for the permeability grade have no obvious difference when some important factors are missing. For instance, although the CRR of the 5–10 mm ceramsite aggregates is not considered in model 43, the *R*^2^ of model 43 is still higher than 0.85; however, the CRR of the 5–10 mm ceramsite aggregates plays an important role in the permeability grade according to the analysis of orthogonal method. This is obviously inconsistent and unreasonable. By contrast, for the residual strength, only two models (60 and 63) present higher values of *R*^2^ (over 0.95). Specially, although the silica fume is not considered in model 60, the effect of silica fume on residual strength is the weakest among all the influencing factors according to the analysis of orthogonal method. It shows that the effectiveness of residual strength is better than that of permeability grade. It is reasonable to speculate that the residual strength may be more suitable for evaluating the permeability characteristics of ceramsite concrete. It also implies that one advantage of the proposed Bayesian framework is beneficial to evaluate the effectiveness of different performance indexes.

In addition, the Log–Ockham factor ranges from −1.96 to 4.87 in the permeability grade candidate models and from −3.15 to 3.54 in the residual strength candidate models. The Log–Ockham factor of model 63 is the highest, indicating that the robustness of model 63 is the best. In other words, the prediction values obtained by model 63 are less sensitive to the errors and noise. Although the results of the previous analysis found that each factor has a different degree of influence through the orthogonal analysis method and the proposed Bayesian framework, the robustness found that each factor has its role. Hence, the model with six factors at the same time is the most stable.

According to the coefficient obtained by the proposed Bayesian method, the recommended prediction equation of permeability grade and residual strength can be expressed by Equations (11) and (12), respectively:

(11) $$P_{N}=0.717+0.661W_{N}+0.089F_{N}-0.258S_{N}+0.176CRR1_{N}-0.489CRR2_{N}-0.106CRR3_{N}$$

(12) $$R_{N}=0.298+0.444W_{N}-0.251F_{N}-0.039S_{N}-0.307CRR1_{N}+0.074CRR2_{N}+0.078CRR3_{N}$$

The above output prediction equation is another advantage of the proposed Bayesian framework compared with the orthogonal method. In the orthogonal method, some factors are difficult to evaluate for their synthetical effect (positive or negative) on the performance, such as Figure 5e,f and Figure 6e,f. The proposed Bayesian framework is conducive to visually observing the influence degree and trend of various factors on the performance. A positive correlation means that one input factor (e.g., *W_N_*, *F_N_*, …) increases as the output index (i.e., *P_N_* and *R_N_*) increases. A negative correlation is when an increase in one input factor causes a decrease in output index. Owing to all variable factors being normalized, the fitting coefficient in Equations (11) and (12) can be regarded as the influence weight related to the variables pertinent. For instance, (a) the coefficient of the water–binder ratio (*W_N_*) in permeability grade and residual strength is the highest, indicating that the water–binder ratio has the most significant influence on the permeability grade and residual strength and can improve the permeability characteristics; (b) the coefficient of silica fume (*S_N_*) in residual strength is negative and the lowest, indicating that the silica fume has the weakest influence on residual strength and will weaken the concrete performance. In Equations (11) and (12), the influence weights and the positive–negative correlations of the main factors are basically consistent with the results obtained from the orthogonal method, such as the water–binder ratio (*W_N_*), the 10–20 mm ceramsite aggregates (CRR2*_N_*) for the permeability grade, and the 5–10 mm ceramsite aggregates (CRR1*_N_*) for the residual strength. It also demonstrates the reliability and feasibility of the proposed Bayesian framework. In addition, when it is time to simplify parameters and increase efficiency, some weaker factors can be accurately eliminated through the Bayesian approach, such as model 60 for the residual strength. The *R*^2^ is higher than 0.95 without the factor of silica fume.

Finally, it only takes less than 5 s to complete the whole analysis process using the proposed Bayesian framework, which can save time compared to orthogonal analysis processing. The advantage will be more obvious with the increase in the content of data. Moreover, ANOVA and regression analysis are also beneficial to quantifying the effect of each factor on permeability resistance, which will be addressed in our future studies.

## 5. Conclusions

In this study, the effects of ceramsite content and size on permeability characteristics are investigated from two aspects: permeability grade and residual compressive strength after permeability. Moreover, an improved Bayesian framework for small sample data was established to analyze the permeability grade and residual strength to estimate these factors more comprehensively. The following conclusions can be drawn:

- The water–binder ratio and the 20–30 mm ceramsite aggregates are the most and least significant factors affecting the permeability grade and residual strength, respectively. The 5–10 mm and 10–20 mm ceramsite aggregates play secondary roles. Moreover, the influence of the 5–10 mm and 10–20 mm ceramsite aggregates on the residual strength is weaker than that on the permeability grade.
- The water–binder ratio is positively correlated with the permeability characteristics, while the 5–10 mm and 10–20 mm ceramsite aggregates present a negative effect; although, 10–20 mm ceramsite aggregates can improve the residual strength when they reach a certain content. Hence, the content of the 5–10 mm and 10–20 mm ceramsite aggregates should be controlled during ceramsite concrete design, and the content of 20–30 mm ceramsite aggregates can be appropriately increased to reduce concrete self-weight without weakening permeability resistance.
- An improved Bayesian framework for small sample data is proposed to analyze the effects of various factors on concrete permeability characteristics. The results obtained by the proposed Bayesian framework and the orthogonal analysis method have good consistency. It demonstrates the reliability and feasibility of the proposed Bayesian framework.
- The proposed Bayesian framework can cut the time cost and provide parameter visualization for the analysis process, compared with the orthogonal method. It is also beneficial in evaluating the effectiveness of different performance indexes. The residual strength may be more suitable for evaluating the permeability resistance than the permeability grade.

## Figures and Tables

**Table 1:** Physical properties of cement.

Index		Result
Al_2_O_3_ content (%)		6.27
Fe_2_O_3_ content (%)		2.79
SiO_2_ content (%)		20.60
K_2_O content (%)		0.67
SO_3_ content (%)		2.24
CaO content (%)		58.50
MgO content (%)		3.98
Cl^−^ content (%)		0.037
Firing loss (%)		3.26
Fineness (%)		5.9
Standard consistency (%)		28.3
Setting time (min)	Initial setting	215
	Final coagulation	275
Stability	Cake test	Qualified
Flexural strength (MPa)	3-day	5.7
Compressive strength (MPa)		26.8

**Table 2:** Technical indexes of pulverized coal ash ceramsite aggregates.

Index	Result
Packing density (kg/m^3^)	1030
Apparent density (kg/m^3^)	1730
Cylinder compressive strength (MPa)	16.4
Water absorption (%/h)	9.8
Mud content (%)	1.0
Chloride (measured by chloride ion content) content (%)	0.01

**Table 3:** Technical properties of aggregates [30].

Index	Result
Crushed stone value (%)	14.4
Los Angeles abrasion value (%)	16.2
Ruggedness (%)	6.2
Flat-elongated particles content (%)	6.1
<0.075 mm particle content (%)	0.5
Water absorption (%)	0.8

**Table 4:** Technical properties of river sand.

Index	Result
Modulus of fineness	2.7
Packing density (kg/m^3^)	1392
Apparent density (kg/m^3^)	2588
Particle size (mm)	<5

**Table 5:** Technical properties of fly ash.

Physical Properties	Result	Chemical Composition	Result
Density (kg/m^3^)	2100	SiO_2_	40
Particle size (mesh number)	400	Al_2_O_3_	30
Specific surface area (g/m^2^)	3500	Fe_2_O_3_	4.2
Water absorption (%)	106	CaO	10
Water requirement	≤100%	MgO	2.5
Water content	≤0.9%	K_2_O	1.1

**Table 6:** Technical properties of silica fume.

Index	Result
Density (kg/m^3^)	2200
Activity index (%)	108
Specific surface area (m^2^/g)	20,000
Water demand ratio (%)	123
Silica (%)	92
Total alkali content (%)	0.23
Chloride ion (%)	0.005
SiO_2_	92
SO_3_	0.33

**Table 7:** Technical properties of water-reducing agent.

Index		Result
Water reduction rate (%)		35
Bleeding rate (%)		29
Gas content (%)		4.0
Difference in setting time (min)	Initial setting	+20
	Final coagulation	−30
Compressive strength ratio (%)	1-day	220
	3-day	183
	7-day	180
	28-day	165

**Table 8:** Experimental factors and levels.

Level	Water–Binder Ratio	Fly Ash	Silica Fume	CRR		
				5–10 mm	10–20 mm	20–30 mm
I	0.24	0%	0%	0%	0%	0%
II	0.26	10%	5%	10%	10%	15%
III	0.28	20%	10%	20%	20%	30%
IV	0.30	30%	15%	30%	30%	45%
V	0.32	40%	20%	40%	40%	60%

**Table 9:** The experimental sequence of the orthogonal experiments.

No.	Water–Binder Ratio	Fly Ash	Silica Fume	CRR			Orthogonal Combination
				5–10 mm	10–20 mm	20–30 mm	
1	0.24	0%	0%	0%	0%	0%	A1B1C1D1E1F1
2	0.24	10%	5%	10%	10%	15%	A1B2C2D2E2F2
3	0.24	20%	10%	20%	20%	30%	A1B3C3D3E3F3
4	0.24	30%	15%	30%	30%	45%	A1B4C4D4E4F4
5	0.24	40%	20%	40%	40%	60%	A1B5C5D5E5F5
6	0.26	0%	5%	20%	30%	60%	A2B1C2D3E4F5
7	0.26	10%	10%	30%	40%	0%	A2B2C3D4E5F1
8	0.26	20%	15%	40%	0%	15%	A2B3C4D5E1F2
9	0.26	30%	20%	0%	10%	30%	A2B4C5D1E2F3
10	0.26	40%	0%	10%	20%	45%	A2B5C1D2E3F4
11	0.28	0%	10%	40%	10%	45%	A3B1C3D5E2F4
12	0.28	10%	15%	0%	20%	60%	A3B2C4D1E3F5
13	0.28	20%	20%	10%	30%	0%	A3B3C5D2E4F1
14	0.28	30%	0%	20%	40%	15%	A3B4C1D3E5F2
15	0.28	40%	5%	30%	0%	30%	A3B5C2D4E1F3
16	0.30	0%	15%	10%	40%	30%	A4B1C4D2E5F3
17	0.30	10%	20%	20%	0%	45%	A4B2C5D3E1F4
18	0.30	20%	0%	30%	10%	60%	A4B3C1D4E2F5
19	0.30	30%	5%	40%	20%	0%	A4B4C2D5E3F1
20	0.30	40%	10%	0%	30%	15%	A4B5C3D1E4F2
21	0.32	0%	20%	30%	20%	15%	A5B1C5D4E3F2
22	0.32	10%	0%	40%	30%	30%	A5B2C1D5E4F3
23	0.32	20%	5%	0%	40%	45%	A5B3C2D1E5F4
24	0.32	30%	10%	10%	0%	60%	A5B4C3D2E1F5
25	0.32	40%	15%	20%	10%	0%	A5B5C4D3E2F1

**Table 10:** The results of the orthogonal experiments.

No.	Osmotic Pressure (Mpa)	Permeability Grade	Residual Strength (Mpa)
1	1.3	12	38.4
2	1.3	12	33.4
3	0.5	4	29.0
4	0.6	4	30.8
5	0.6	4	29.5
6	0.5	4	41.6
7	0.6	4	42.4
8	0.5	4	34.8
9	1.1	10	35.9
10	1.3	12	34.4
11	1.3	12	36.3
12	1.3	12	44.4
13	1.3	12	39.7
14	0.8	6	37.2
15	1.3	12	42.2
16	0.8	6	50.2
17	1.2	10	44.0
18	1.3	12	39.2
19	1.3	12	33.7
20	1.3	12	40.1
21	1.3	12	37.7
22	1.3	12	42.6
23	1.3	12	52.3
24	1.3	12	42.3
25	1.3	12	41.8

**Table 11:** The range of the orthogonal experiments.

Performance Indicator			Water–Binder Ratio	Fly Ash	Silica Fume	CRR		
Property	Value	Test Level				5–10 mm	10–20 mm	20–30 mm
Permeability grade	Average value	I	7.2	9.2	10.8	11.6	10	10.4
		II	6.8	10	10.4	10.8	11.6	9.2
		III	10.8	8.8	8.8	7.2	10.4	8.8
		IV	10.4	8.8	7.6	8.8	8.8	10
		V	12	10.4	9.6	8.8	6.4	8.8
	Range		5.2	1.6	3.2	4.4	5.2	1.6
Residual strength	Average value	I	32.24	40.85	38.36	42.22	40.35	39.20
		II	37.83	41.37	40.64	40.02	37.32	36.63
		III	39.96	39.01	38.03	38.72	35.82	39.98
		IV	41.43	35.98	40.40	38.46	38.97	39.57
		V	43.33	37.58	37.36	35.38	42.33	39.41
	Range		11.10	5.39	3.29	6.84	6.51	3.35

**Table 12:** The 63 model classes and the corresponding variables.

Model Class ID	Selected Variables	Variables Number	Model Class ID	Selected Variables	Variables Number
1	W	1	33	F, S, CRR2	3
2	F		34	F, S, CRR3	
3	S		35	F, CRR1, CRR2	
4	CRR1		36	F, CRR1, CRR3	
5	CRR2		37	F, CRR2, CRR3	
6	CRR3		38	S, CRR1, CRRR2	
7	W, F	2	39	S, CRR1, CRR3	
8	W, S		40	S, CRR2, CRR3	
9	W, CRR1		41	CRR1, CRR2, CRR3	
10	W, CRR2		42	W, F, S, CRR1	4
11	W, CRR3		43	W, F, S, CRR2	
12	F, S		44	W, F, S, CRR3	
13	F, CRR1		45	W, F, CRR1, CRR2	
14	F, CRR2		46	W, F, CRR1, CRR3	
15	F, CRR3		47	W, F, CRR2, CRR3	
16	S, CRR1		48	W, S, CRR1, CRR2	
17	S, CRR2		49	W, S, CRR1, CRR3	
18	S, CRR3		50	W, S, CRR2, CRR3	
19	CRR1, CRR2		51	W, CRR1, CRR2, CRR3	
20	CRR1, CRR3		52	F, S, CRR1, CRR2	
21	CRR2, CRR3		53	F, S, CRR1, CRR3	
22	W, F, S	3	54	F, S, CRR2, CRR3	
23	W, F, CRR1		55	F, CRR1, CRR2, CRR3	
24	W, F, CRR2		56	S, CRR1, CRR2, CRR3	
25	W, F, CRR3		57	W, F, S, CRR1, CRR2	5
26	W, S, CRR1		58	W, F, S, CRR1, CRR3	
27	W, S, CRR2		59	W, F, S, CRR2, CRR3	
28	W, S, CRR3		60	W, F, CRR1, CRR2, CRR3	
29	W, CRR1, CRR2		61	W, S, CRR1, CRR2, CRR3	
30	W, CRR1, CRR3		62	F, S, CRR1, CRR2, CRR3	
31	W, CRR2, CRR3		63	W, F, S, CRR1, CRR2, CRR3	6
32	F, S, CRR1				

**Table 13:** Ranges of priors.

Variables	x_0_	W_N_	F_N_	S_N_	CRR1_N_	CRR2_N_	CRR3_N_
Priors	(−1, 1)	(−1, 1)	(−1, 1)	(−1, 1)	(−1, 1)	(−1, 1)	(−1, 1)

## Data Availability

The data presented in this study are available in article.

## References

[1] Yang Q., Yang S.T., Liu Q., Jin L.L. (2024). Study on bond properties between near-surface mounted BFRP bars and concrete under freeze-thaw cycles in seawater. Ocean Eng..

[2] Deng P., Cong Z.R., Liu Y., Huang Y.Y., Zhu Q. (2022). Effect of dry-wet cycles on BFRP bars and modified ceramsite concrete in marine environments. J. Mater. Civil. Eng..

[3] Li P., Li J., Fan L., Mi S.D., Li J.Y., Liu H.Q., Peng S.Q., Huang W.Q. (2024). Experimental investigation into lightweight high strength concrete with shale and clay ceramsite for offshore structures. Sustainability.

[4] Li X., Zhu H.B., Fu Z.H., Liu P., Xia C.H. (2022). Influence of volume-to-surface area ratio on the creep behavior of steel fiber ceramsite concrete beams. Coatings.

[5] Liu P., Luo A., Liu L., Li Y.L., Zhang S.L., Zhi W.T., Pan D., Chen Y., Yu Z.W. (2023). Study on the preparation and performances analysis of lightweight high strength ceramsite aerated concrete. J. Mater. Res. Technol..

[6] Huang C.H., Yuan N.N., He X.S., Wang C.H. (2023). Ceramsite made from drinking water treatment residue for water treatment: A critical review in association with typical ceramsite making. J. Environ. Manag..

[7] Zhu H.B., Xiao Y., Li X., Wang Y., Wang S.Y. (2023). Study on Flexural Strength of Interface between Full Lightweight Ceramsite Concrete and Ordinary Concrete. Coatings.

[8] Zang J.W., Pan C.G., Hu Y., Qu S.Y. (2023). Preparation of ceramsite using dehydrated silt soil and its performance on compressive strength of ceramsite concrete block. Sustainability.

[9] Chen Y., Hui Q.J., Zhang H.W., Zhu Z.J., Wang C.W., Zhao J. (2020). Experiment and application of ceramsite concrete used to maintain roadway in coal mine. Meas. Control.

[10] Han R., Xu Z., Qing Y. (2017). Study on the material performance of ceramsite concrete roof brick. Procedia Eng..

[11] Bu C.M., Zhu D.X., Lu X.Y., Liu L., Sun Y., Yu L.W., Zhang W.T., Xiao T. (2023). Optimization of the water-cement ratio of rubberized ceramsite concrete. J. Rubber Res..

[12] Zhu H.B., Chen J.Y., Wu Y.X., Li J.P., Fu Z.H., Liu P. (2023). Experimental study on flexural bearing characteristics of ceramsite concrete beams after creep at different curing ages. Coatings.

[13] Wu X.G., Wang S.R., Yang J.H., Zhao J.Q., Chang X. (2022). Damage characteristics and constitutive model of lightweight shale ceramsite concrete under static-dynamic loading. Eng. Fract. Mech..

[14] Wang S.R., Wu X.G., Yang J.H., Zhu S. (2020). Acoustic emission characteristics and dynamic damage constitutive relation of shale-ceramsite concrete subjected to loading tests. J. Mater. Civil. Eng..

[15] De Maio U., Greco F., Lonetti P., Pranno A. (2024). A combined ALE-cohesive fracture approach for the arbitrary crack growth analysis. Eng. Fract. Mech..

[16] Yuan P., Zhu Y.S., Li D.H., Lu X.F. (2024). Effect of freeze-thaw cycle and moisture content on compressive and energy properties of alkali slag ceramsite concrete. KSCE J. Civ. Eng..

[17] Zeng Y.S., Zhou X.Y., Tang A.P. (2021). Shear performance of fibers-reinforced lightweight aggregate concrete produced with industrial waste ceramsite-Lytag after freeze-thaw action. J. Clean. Prod..

[18] Zeng Y.S., Meng S.H., Xu H.Y., Yuan S.C., Lang W., Chen W. (2022). Strength attributes of fiber-reinforced lightweight aggregate concrete incorporating Lytag ceramsite under freeze-thaw environment. J. Build. Eng..

[19] Shen Y., Ma X., Huang J., Hao F., Lv J., Shen M. (2020). Near-zero restrained shrinkage polymer concrete incorporating ceramsite and waste rubber powder. Cement Concrete Comp..

[20] Zhang Y.T., Sun X.W. (2023). Synergistic effects of nano-silica and fly ash on the mechanical properties and durability of internal-cured concrete incorporating artificial shale ceramsite. J. Build. Eng..

[21] Yao W., Pang J., Liu Y. (2020). Performance degradation and microscopic analysis of lightweight aggregate concrete after exposure to high temperature. Materials.

[22] Zhang D., Yang W. (2015). Test research on bond behaviors between shale ceramsite lightweight aggregate concrete and bars through pullout tests. J. Mater. Civil. Eng..

[23] Gao S., Huang K., Chu W., Wang W. (2023). Feasibility study of pervious concrete with ceramsite as aggregate considering mechanical properties, permeability, and durability. Materials.

[24] Fan L., Zhang Z.J., Yu Y.Q., Li P.T., Cosgrove T. (2017). Effect of elevated curing temperature on ceramsite concrete performance. Constr. Build. Mater..

[25] Palanisamy M., Kolandasamy P., Awoyera P., Gobinath R., Muthusamy S., Krishnasamy T.R., Viloria A. (2020). Permeability properties of lightweight self-consolidating concrete made with coconut shell aggregate. J. Mater. Res. Technol..

[26] Bulut H.A. (2023). Examination of mechanical; permeability, and durability properties of sustainable lightweight concrete composites with natural perlite aggregate. IJST-T. Civ. Eng..

[27] Chao Z.M., Wang H.Y., Hu S.Y., Wang M., Xu S.K., Zhang W.B. (2024). Permeability and porosity of light-weight concrete with plastic waste aggregate: Experimental study and machine learning modelling. Constr. Build. Mater..

[28] Kong L.J., Du Y.B. (2015). Effect of lightweight aggregate and the interfacial transition zone on the durability of concrete based on grey correlation. Indian J. Eng. Mater. S.

[29] Chung S.Y., Sikora P., Kim D.J., El Madawy M.E., Elrahman M.A. (2021). Effect of different expanded aggregates on durability-related characteristics of lightweight aggregate concrete. Mater. Charact..

[30] Ren J.L., Li D., Xu Y.S., Huang J.D., Liu W. (2021). Fatigue behaviour of rock asphalt concrete considering moisture, high-temperature, and stress level. Int. J. Pavement. Eng..

[31] (2020). Testing Method of Cement and Concrete for Highway Engineering.

[32] Zeng J.C., Kim Y.H., Qin S.Q. (2023). Bayesian model updating for structural dynamic applications combing differential evolution adaptive metropolis and kriging model. J. Struct. Eng..

[33] Yan W.M., Yuen K.V., Yoon G.L. (2009). Bayesian probabilistic approach for the correlations of compression index for marine clays. J. Geotech. Geoenviron. Eng..

[34] Papadimitriou C., Beck J.L., Katafygiotis L.S. (1997). Asymptotic expansions for reliabilities and moments of uncertain dynamic systems. J. Eng. Mech..

[35] Beck J.L., Yuen K.V. (2004). Model selection using response measurements: Bayesian probabilistic approach. J. Eng. Mech..

